# Risk factors for depth of infiltration in the differentiated depressed early gastric carcinoma: a preliminary analysis

**DOI:** 10.1186/s13000-014-0206-8

**Published:** 2014-10-30

**Authors:** Shu-dong Yang, Zhi-xin Qian, Qiang Zhan, Qun-yan Zhou, Guo-min Lu

**Affiliations:** Department of Pathology, Wuxi People’s Hospital Affiliated with Nanjing Medical University, Wuxi, 214023 China; Department of Internal Medicine, Jiangsu Taizhou People’s Hospital, Taizhou, 225300 China; Department of Gastroenterology, Wuxi People’s Hospital Affiliated with Nanjing Medical University, Wuxi, 214023 China

**Keywords:** Differentiated depressed early gastric cancer, Depth of infiltration, Clinicopathologic factor

## Abstract

**Background:**

To analyze the clinicopathologic factors associated with mucosal and submucosal infiltration in differentiated depressed early gastric cancer, and screening factors that can predict depth of infiltration before endoscopic treatment.

**Methods:**

The study included 35 cases of mucosal carcinomas and 66 cases of submucosal carcinomas according to the pathological diagnosis. The relevant clinicopathologic factors were investigated by univariate and multivariate analysis.

**Results:**

The average depth of the depressed lesions for the submucosal group was significantly more than that for the mucosal group. The proportion of the lesions with rough bottom surface and abnormal surrounding folds was significantly higher in the submucosal group compared to that in the mucosal group. Logistic regression analysis indicated that the above-mentioned three factors were independent risk factors that could be used to predict mucosal and submucosal infiltration. Area under the curve (AUC) of receiver operating characteristic (ROC) of the ordinal above-mentioned three factors for predicting submucosal infiltration was 0.716, 0.663, 0.704, respectively. Stratified analysis showed that the 100% cases with lesion depth ≥2.5 mm and rough bottom surface developed submucosal infiltration regardless of the morphological changes of the folds.

**Conclusion:**

The study identified independent risk factors for predicting mucosal and submucosal infiltration in depressed differentiated early gastric cancer, which may evaluate the degree of penetration before endoscopic treatment.

**Virtual Slides:**

The virtual slide(s) for this article can be found here: http://www.diagnosticpathology.diagnomx.eu/vs/13000_2014_206

## Background

Endoscopic mucosal resection (EMR) and endoscopic submucosal dissection (ESD) have become one of the standard treatment methods for early-stage gastric cancer in Japan. The development of these two endoscopic technologies has benefited from research on two screening criteria for operability for endoscopic resection. Yamao et al. [1] and Gotoda et al [2], investigated thousands of patients with early gastric cancer who received gastrectomy and lymph node dissection, and assessed the clinicopathological factors for their possible association with lymph node metastasis. They eventually developed evaluation criteria for EMR and ESD treatment of early gastric cancer before surgery.

EMR and ESD are therapeutic endoscopic techniques that offer the advantage of acquiring specimens from the lesion for pathological analysis. These have become the most accurate approaches for the diagnosis of early gastric cancer. For instance, results of the pathological examination can confirm the cure or suggest additional treatment. The disease-specific 5- and 10-year survival rates can reach 99% for patients who meet the above evaluation criteria for EMR treatment of early gastric cancer before surgery [3].

Early gastric ESD offers the advantage of high percentage of enbloc resection rates. According to a published report, enbloc resection rates of 92.7% and curative resection rates of 73.6% were achieved [4]. Local recurrence of early gastric cancer after ESD treatment was less than 2% [5]. It is worth noting that the healing effects similar to those obtained with traditional surgical treatment could be achieved only if the lesions met the criteria for curative resection, highlighting the importance of accurate pre and post-operative assessment. The mistakes in preoperative evaluation do occur (error rate was 7% in the EMR group and 16% in the ESD group) and are confirmed by postoperative pathological examination.4 According to preoperative indications defined by Japanese Gastric Cancer Association (JGCA) [6,7], endoscopic examination and endoscopic biopsy are sufficient to evaluate the diameter of the lesion, formation of ulcer and the pathological type of early gastric cancer. However, practical and putative criteria to evaluate the depth of mucosal or submucosal infiltration are still lacking.

Given the difficulty in determining the depth of infiltration in the depressed early gastric cancer, we followed the method of Yamao T and Gotoda T [1,2], and retrospectively reviewed the clinicopathological characteristics of mucosal and submucosal gastric cancer in order to identify the factors for preoperative evaluation in gastric cancer.

## Methods

### Subjects and inclusion/exclusion criteria

Patients who had been diagnosed for depressed early gastric cancer at Wuxi People’s Hospital Affiliated with Nanjing Medical University between January 2005 and December 2011 were recruited for this study. The subjects received surgical resection and lymphadenectomy and the pathological analysis confirmed diagnosis of differentiated type early gastric cancer. Cases with undifferentiated type early gastric cancer were excluded due to the controversy surrounding the use of endoscopy for such types of gastric cancer. All case diagnoses were made by a pathologist with 16 years clinical experience and confirmed by an independent pathologist with over 40 years clinical experience. Informed consents were obtained from all subjects, and the study was approved by the medical ethics committee of People’s Hospital of Wuxi, an affiliated hospital of Nanjing Medical University.

### Test parameters

According to the classification of gastric cancer based on the guidelines laid out by JGCA [7], the lesions were grouped as: type I (elevated), type II and type III (depressed). Type II can be sub-classified into: type IIa (surface elevated), type IIb (flat) and type IIc (surface depressed). Both type III and type IIc were considered as depressed lesions.

The following clinical data were acquired: (1) gender; (2) age; (3) location of the lesions (stomach was divided into upper 1/3, middle 1/3 and lower 1/3 based on JGCA guidelines;7 (4) size of the lesion (largest diameter of the bottom surface of depressed area); (5) presence of ulcer (obvious ulcer or scarring evidence of a previous ulcer); (6) depth of the lesion (measured as the distance between planes of the specimen mounted on a pathology slide from the highest point of the lesion edge to the lowest point of the lesion basilar portion); (7) bottom of the lesion: smooth or rough (surface is not flat with particle-like bulge or small mucosal island); (8) folds surrounding the lesions: normal folds were marked as negative and broken folds or enlarged/fusion folds were marked as positive; (9) degree of gastric cancer differentiation: highly differentiated was defined as grade I cancer and moderately differentiated was defined as grade II cancer; (10) depth of infiltration: the lesions were classified as mucosal gastric cancer or submucosal gastric cancer.

### Statistical analysis

The data were processed using SPSS 17.0 software (SPSS Inc., Chicago, IL). All the samples were classified into the mucosal group and the submucosal group based on the depth of infiltration. T test or Wilcoxon rank test were used to process quantitative data, and categorical data were processed by chi-square test. Logistic regression multiple factor analysis was used to evaluate independent factors that influenced the mucosal/submucosal infiltration of early gastric cancer. Receiver Operating Characteristic (ROC) curves of independent factors were plotted based on the above results of Logistic regression analysis to reflect morphological factors, which may affect the depth of infiltration. The area under the curve (AUC) was calculated and cut-off values were estimated. Stratified analysis was performed for statistically significant morphological factors to evaluate the distribution of mucosal and submucosal groups in various conditions. P < 0.05 was defined as statistically significant.

## Results

**Figure 1:**
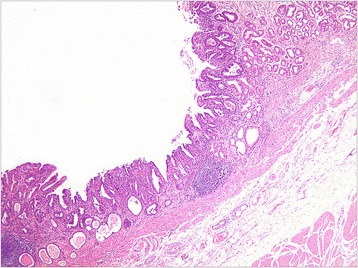
**A representative moderately differentiated adenocarcinoma with invasion of the muscular layer of mucosa.** HE staining, low power (40×) microscopic view.

**Figure 2:**
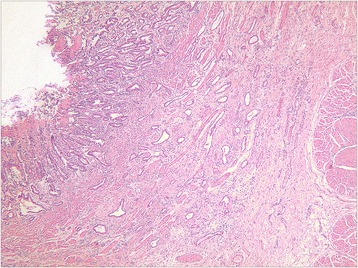
**A representative moderately differentiated adenocarcinoma with invasion of the submucosa.** HE staining, low power (40×) microscopic view.

A total of 101 cases with depressed lesions were evaluated (35 cases in the mucosal group, 34.7%, Figure 1; 66 cases in the submucosal group, 65.3%, Figure 2). General patient information and clinicopathologic data are shown in Tables 1 and 2.

**Table 1:** **General patient information**

	**N =101**			
**Patient data**	**Males, N = 71**		**Females, N = 30**	
	Yes	No	Yes	No
Upper abdominal pain	50	21	19	11
Abdominal distention	41	30	19	11
Decreased food appetite	16	55	5	25
Nausea	25	46	11	19
Vomiting	3	68	3	27
Acid regurgitation	5	66	3	27
Belching	16	55	14	16
Haematemesis	5	66	0	30
Black stool	14	57	0	30
Weight loss	9	62	3	27
Fatigue	7	64	3	27
History of hypertension	27	44	16	14
History of coronary heart disease	5	66	0	30
History of diabetes mellitus	11	60	8	22
History of hepatitis	5	66	0	30
History of cirrhosis	3	68	0	30
Long-term* history of smoking	13	58	0	30
Long-term** history of drinking alcohol	3	68	0	30
Long-term*** oral administration of aspirin	3	68	0	30
Special occupation	3****	68	0	30
Family history	5	66	3	27

*15-30 years; **7-15 years; ***3-5 years; ****All cases were dust-exposure.

**Table 2:** **Clinical and pathological characteristics of the patients**

**Characteristic**		**N**	**Percentage (%)**
Sex	Male	71	70.3
	Female	30	29.7
Age in years		61.2 ± 9.8	
Location of lesions	Upper 1/3	24	23.8
	Middel 1/3	30	29.7
	Lower 1/3	47	46.5
Size of the lesions in mm	15.5 ± 12.3		
Presence of ulcer	No	53	52.5
	Yes	48	47.5
Depth of depressed lesions in mm	3.4 ± 2.4		
Roughness of the bottom	Smooth	65	64.4
	Rough	36	35.6
Surrounding folds	Normal	51	50.5
	Abnormal	50	49.5
Degree of differentiation	Highly differentiated	10	9.9
	Moderately differentiated	91	91.1

Data are presented as N or mean ± SD.

### Univariate analysis of the depressed lesions

Univariate analysis was applied to the data of acquired characteristics. T test or Wilcoxon rank test were used to process quantitative data, and categorical data were processed by chi-square test. Results showed that the lesion depth of the submucosal group (average 3.9 ± 2.5 mm) was more than that of the mucosal group (average 2.3 ± 1.5 mm). The analysis of the bottom of the lesion showed that the mucosal group had 30 (85.7%) cases with smooth bottom and 5 (14.3%) cases with rough bottom, while the submucosal group had 35 (53.0%) cases of smooth bottom and 31 (47.0%) cases of rough bottom. The analysis of folds surrounding the lesions showed that the mucosal group had 27 (77.1%) cases with normal folds and 8 (22.9%) cases with abnormal folds, while the submucosal group had 24 (36.4%) cases with normal folds and 42 (63.6%) cases with abnormal folds. The submucosal group showed higher rates of rough bottom surface and abnormal folds compared to the mucosal group. The collective data is presented in Table 3.

**Table 3:** **Univariate analysis of differentiated depressed early gastric carcinoma**

**Factors**		**Values**		**Statistical value**	***p value***
		**Mucosal gastric cancer**	**Submucosal gastric cancer**		
Sex	Male	24 (66.2)	47 (71.2)	χ^*2*^ = 0.076	0.782
	Female	11 (33.8)	19 (28.8)		
Age in years		62.3 ± 9.2	60.6 ± 10.2	*t* = 0.402	0.843
Lesion location	Upper 1/3	6 (17.1)	18 (27.3)	χ^*2*^ = 1.994	0.369
	Middle 1/3	13 (37.2)	17 (25.7)		
	Lower 1/3	16 (45.7)	31 (47.0)		
Size of the lesion in mm		12.4 ± 10.8	17.2 ± 12.8	*U* = 894.0	0.061
Ulcer	Absence	22 (62.9)	31 (47.0)	χ^*2*^ = 2.315	0.128
	Presence	13 (37.1)	35 (53.0)		
Depth in mm		2.3 ± 1.5	3.9 ± 2.5	*U* = 656.5	0.000
Roughness of the bottom	Smooth	30 (85.7)	35 (53.0)	χ^*2*^ = 10.651	0.001
	Rough	5 (14.3)	31 (47.0)		
Surrounding folds	Normal	27 (77.1)	24 (36.4)	χ^*2*^ = 15.215	0.000
	Abnormal	8 (22.9)	42 (63.6)		
Degree of differentiation	Highly differentiated	4 (11.4)	6 (9.1)	χ^*2*^ = 0.140	0.708
	Moderately differentiated	31 (88.6)	60 (90.9)		

Data are presented as N (%) or mean ± SD.

### Multivariate analysis of depressed lesions

According to the results of univariate analysis, logistic binary regression model was used to evaluate the size, depth, bottom surface, and surrounding folds of the lesions (P < 0.1). Regression model was established using forward stepwise regression, and the results obtained from the three-step regression analysis showed that depth, surface roughness and surrounding folds of the lesions are independent predictors of tumor filtration (Table 4).

**Table 4:** **Multivariate analysis of differentiated depressed early gastric carcinoma**

	**B**	**S.E.**	**Wals**	**df**	**Sig.**	**Exp (B)**	**Exp (B) 95% CI**	
							**Lower limit**	**Upper limit**
Lesion depth	0.425	0.151	7.949	1	0.005	1.530	1.138	2.056
Bottom roughness	1.348	0.608	4.918	1	0.027	3.851	1.170	12.681
Surrounding folds	1.448	0.520	7.746	1	0.005	4.254	1.535	11.794

*Abbreviations*: *B* regression coefficient, *S.E.* standard error, *Wals* the square of the ratio of the regression coefficient and standard error, *df* degrees of freedom, *Sig* probability, *Exp (B)* the exponential function, and is the result of regression coefficient B value index calculation, *CI* confidence interval.

### Diagnostic performance of independent morphological factors of the depressed lesions for predicting submucosal infiltration

**Figure 3:**
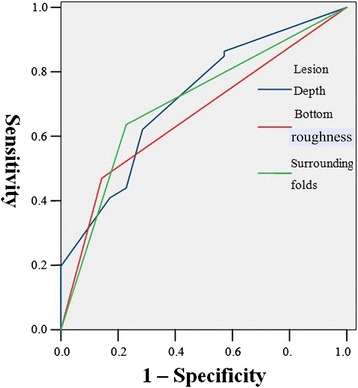
**ROC curve to predict infiltration depth based on lesion depth, bottom roughness and surrounding folds.**

We further plotted ROC curves to estimate the diagnostic performance of the three independent predictors: depth, surface roughness and surrounding folds of the depressed lesions in gastric cancer (Figure 3). AUCs of depth, surface roughness and surrounding folds of the lesions were 0.716 (P = 0.000, 95% CI 0.613-0.819), 0.663 (P = 0.007, 95% CI 0.556-0.771) and 0.704 (P = 0.001, 95% CI 0.598-0.810), respectively. The optimal cut-off value of depth of depressed lesions for predicting submucosal infiltration was 2.5 mm based on Youden’s index (Table 5).

**Table 5:** **ROC curve and Youden’s index based on the depth of the lesions**

**Lesion depth**	**Sensitivity**	**Specificity**	**1—specificity**	**Youden’s index** ^**a**^
0.000	1.000	0.000	1.000	0.000
1.250	0.864	0.429	0.571	0.293
1.750	0.848	0.429	0.571	0.277
2.500^b^	0.621	0.714	0.286	0.315
3.500	0.439	0.771	0.229	0.210
4.500	0.409	0.829	0.171	0.238
5.500	0.197	1.000	0.000	0.197
6.000	0.167	1.000	0.000	0.167
7.500	0.121	1.000	0.000	0.121
9.000	0.061	1.000	0.000	0.061
11.000	0.000	1.000	0.000	0.000

^a^Youden’s index = (sensitivity + specificity)-1; ^b^the optimal cut-off value.

### Stratified analysis

Based on the cut-off value of 2.5 mm, the lesions were divided into two groups: <2.5 mm group and ≥2.5 mm group. Stratified analysis suggested that 100% of the lesions in early, depressed gastric cancer with depth ≥2.5 mm, rough bottom surface and normal/abnormal surrounding folds developed submucosal infiltration (Table 6).

**Table 6:** **Stratified analysis of the differentiated depressed early gastric carcinoma**

**Lesion depth**	**Bottom roughness**				**Grouping based on penetration depth**		**Sum**
					**Mucosal surface**	**Submucosal surface**	
< 2.5 mm	Smooth	Surrounding folds	No	Count	16	7	23
				Percentage	69.5%	31.5%	100.0%
			Yes	Count	4	6	10
				Percentage	40.0%	60.0%	100.0%
	Rough	Surrounding folds	No	Count	4	4	8
				Percentage	50.0%	50.0%	100.0%
			Yes	Count	1	8	9
				Percentage	11.1%	88.9%	100.0%
≥ 2.5 mm	Smooth	Surrounding folds	No	Count	7	11	16
				Percentage	43.8%	56.2%	100.0%
			Yes	Count	3	11	14
				Percentage	21.4%	79.6%	100.0%
	Rough	Surrounding folds	No	Count	0	2	2
				Percentage	0.0%	100.0%	100.0%
			Yes	Count	0	17	17
				Percentage	0.0%	100.0%	100.0%

## Discussion

Endoscopic mucosal resection (EMR) and endoscopic submucosal dissection (ESD) are widely accepted endoscopic techniques for treating early gastric cancer. The indications for applying endoscopic therapy are: differentiated type early gastric cancer; no evidence of lymph node metastasis; infiltration is confined to mucosal or minute submucosal infiltration [8]. The rate of lymph node metastasis is less than 3% when the gastric cancer is confined to mucosal infiltration [9]. However, submucosal infiltration leads to a higher risk of metastasis [10]. Due to frequent submucosal infiltration and lymph node metastasis [11], evaluation of the depth of infiltration of depressed type of early gastric cancer is crucial for decisions regarding the therapeutic regimen [8,12].

There is no consensus on the preoperative diagnosis of the depth of infiltration. Computed tomography (CT) scan does not assess the depth of tumor invasion [13]. The use of endoscopic ultrasonography is also controversial in determining the depth of infiltration [14]. A summary of 18 studies suggested that sensitivity varied from 18.2% to 100% (average 87.8%) and specificity varied from 34.7% to 100% (average 80.2%) when using endoscopic ultrasound for the diagnosis of submucosal invasion of early gastric cancer [15]. It was concluded that it is not a mature method for differentiating between mucosal and submucosal infiltration. Traditionally, gastroscopy has been used for early gastric cancer staging with an accuracy of 79.0%, which is similar to that achieved by endoscopic ultrasound [16-18]. Thus, ordinary endoscopy could provide sufficient information to determine the best treatment options for the resection of early gastric cancer.

Our study identified independent predictive factors for infiltration based on the analysis of multiple clinical and pathomorphological data. We further applied stratified analysis to these factors to predict the depth of filtration. It was reported that the diameter of the lesion is associated with the depth of infiltration of gastric cancer [19], but our results indicated that there was no significant difference between mucosal and submucosal groups. Besides, only seven cases of depressed lesions among 37 cases of gastric cancer were included [19], which limits the analysis performed in that study. The parameters, such as the location of the lesion, presence of ulcer and histology type are irrelevant to the depth of filtration, which is consistent with a previously published study [20]. ROC curve analysis further illustrates the value of the three factors for the diagnosis of lesion infiltration. Among the three factors, the depth of the lesion is superior to the lesion bottom roughness and surrounding folds in term of its value for making a diagnosis. Our findings that the presence of rough bottom surface and irregular folds also aid in the diagnosis of submucosal gastric cancer are in agreement with a pervious study [21].

It should be noted that in this study, the converging folds were considered as ulcer but not the abnormal changes of the surrounding folds. Only the broken and enlarged folds were defined as abnormal folds. The formation of ulcers and subsequent scarring and converging fold normally indicate tissue fibrosis but do not necessarily suggest penetration of tumor, although some gastric cancers with converging folds are confined to the mucosal layer [22]. However, the folds may break or enlarge when the top of the folds are infiltrated by the tumor. If annular dike appears, the tumor may have invaded the muscular layer. Thus, abnormal folds but not the presence or absence of ulcers may provide useful information for evaluating the depth of penetration in differentiated gastric cancer.

Based on the Youden’s index derived from the ROC curve, we defined 2.5 mm as the cut-off value to divide the lesions into two groups (lesion depth <2.5 mm or ≥2.5 mm). Indeed, cases with lesion depth ≥2.5 mm with rough bottom surface had 100% submucosal penetration, regardless of the status of the surrounding folds. However, 69.5% cases with lesion depth <2.5 mm, smooth bottom and normal surrounding folds were confined to the mucosa. However, we measured the depth using specimens mounted on glass slides for pathological analysis; as such, the distance is expected to have been affected (shrunk by 10-20%) by the formalin fixation and dehydration processes that are required for slide preparation. Our study aimed to reflect the conditions found under endoscopy and before any biopsy is taken. Therefore, the cut-off value of 2.5 mm may actually be 3 mm in living stomach. It is important that any future research studies, either from our lab or others, should design and use measurement methods that will reduce this kind of error.

## Conclusions

Overall, we identified lesion depth, bottom roughness and surrounding folds as independent predictive factors for evaluating mucosal or submucosal infiltration in depressed gastric cancer based on retrospective analysis of the pathology samples. The results indicate that further studies from multiple centers and with larger sample sizes are required.
